# Diffuse Idiopathic Pulmonary Neuroendocrine Cell Hyperplasia: Review of the Literature and a Single-center Experience

**DOI:** 10.7759/cureus.5640

**Published:** 2019-09-13

**Authors:** Luis Cabezón-Gutiérrez, Parham Khosravi-Shahi, Magda Palka-Kotlowska, Sara Custodio-Cabello, Maria García-Martos

**Affiliations:** 1 Medical Oncology, Hospital Universitario De Torrejon, Madrid, ESP; 2 Medical Oncology, Hospital General Universitario Gregorio Marañon, Madrid, ESP; 3 Medical Oncology, Hospital Universitario De Torrejón, Madrid, ESP; 4 Pathology, Hospital Universitario De Torrejón, Madrid, ESP

**Keywords:** diffuse idiopathic pulmonary neuroendocrine cell hyperplasia, tumorlets, carcinoid, case report

## Abstract

Diffuse idiopathic pulmonary neuroendocrine cell hyperplasia (DIPNECH) is a rare disorder that is commonly underdiagnosed. In 2015, it was recognized by the World Health Organization (WHO) classification of lung tumors as a premalignant lesion. DIPNECH syndrome is characterized by cough, exertional dyspnea, wheezing, and, less frequently, hemoptysis. We report the clinical and histological features and imaging findings in four cases of DIPNECH from our institution (Torrejon University Hospital, Madrid, Spain) between the years 2012 and 2019. DIPNECH represents a rare and poorly understood pulmonary disorder. Our limited single-center experience shows the slow and stable evolution of the disease. However, some exceptional cases may progress poorly if distant metastases occur.

## Introduction

Diffuse idiopathic pulmonary neuroendocrine cell hyperplasia (DIPNECH) is a rare disorder that is commonly underdiagnosed. In 2015, it was recognized by the World Health Organization (WHO) classification of lung tumors as a premalignant lesion. Aguayo et al. [1] were the first to describe this entity, showing hyperplasia confined to the respiratory epithelium layer without penetration of the basement membrane. It occurs predominantly in females (ratio 10:1) with a median age of 58 years at diagnosis, and it is not associated with smoking [2]. DIPNECH syndrome is characterized by cough, exertional dyspnea, wheezing, and, less frequently, hemoptysis. Histologically, it manifests as a generalized proliferation of scattered neuroendocrine cells, tiny nodular aggregates, or a linear proliferation of neuroendocrine cells [3].

We report the clinical evolution, histological features, and imaging findings of four cases of DIPNECH diagnosed in our institution (Hospital Universitario de Torrejón, Madrid, Spain) between the years 2012 and 2019.

## Case presentation

Case 1

**Figure 1:**
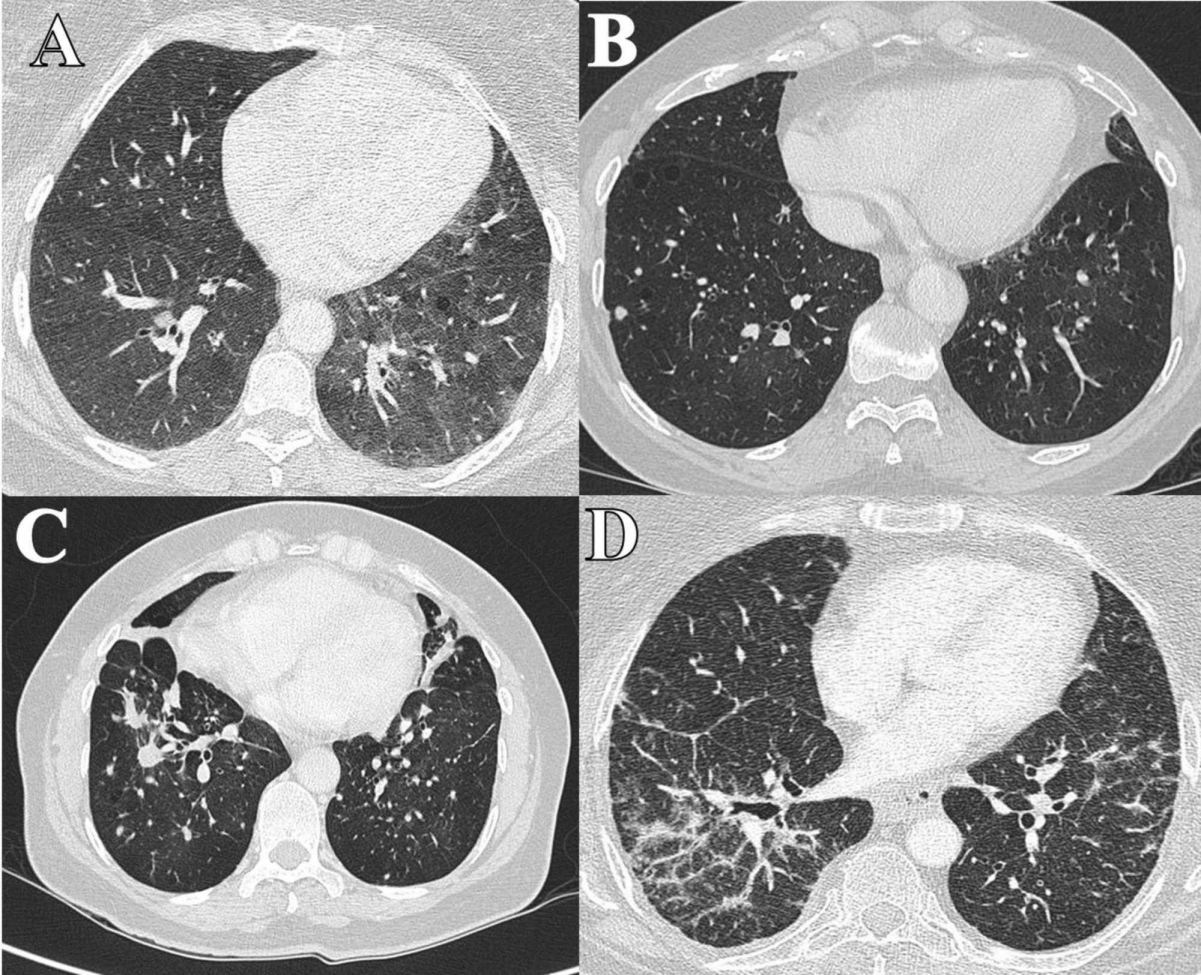
CT images (pulmonary parenchyma window). A (Case 1 patient). B (Case 2 patient). C (Case 3 patient). D (Case 4 patient).

A 48-year-old female patient was admitted in October 2013 to the digestive surgery department of our hospital due to one year of proctalgia and perianal tumor growth. She was a smoker of 40 cigarettes/day and presented non-productive cough and great effort dyspnea. The extension computed tomography (CT) study showed diffuse interstitial pulmonary involvement with multiple bilateral pulmonary nodules, the largest being 1.1 cm in the lower left lobe (LLL) (Figure 1A). Positron emission tomography (PET)-CT showed pathological uptake with a standardized uptake value (SUV) of 2.2 in the nodular lesion of LLL. The others of the subcentimeter nodules did not show glucose uptake. An Indio (111 In) pentetreotide scintigraphy (Octreoscan) was performed, with a negative result. Bronchoscopy did not reveal any endobronchial finding. Chromogranin A (CgA) serum level and 24-hour urine analysis for 5-hydroxyl-indole-acetic (u 5-HIAA) were in the normal range.

She was diagnosed with stage I perianal squamous cell carcinoma, positive human papilloma virus-positive, and negative human immunodeficiency virus-negative. She underwent wide local excision of the anal carcinoma with negative margins. A CT-guided thick-needle biopsy of the LLL lesion was performed and a low-grade neuroendocrine tumor (Ki 67 <1%) was observed. Functional respiratory tests were in the normal range (forced expiratory volume in one second (FEV1) 60%, forced vital capacity (FVC) 54%, and single-breath carbon monoxide diffusing capacity (DLCO) 61%) and did not contraindicate surgery. The patient underwent left lower lobectomy and the dissection of levels 9, 11, 7, and 5 lymph nodes due to the suspicion of DIPNECH with a carcinoid tumor in LLL.

The histological sections showed at the level of the lobar bronchus, as well as in the peripheral lesion of 9 mm, a neoplastic proliferation of epithelial character constituted by a monomorphic population of cells of neuroendocrine habit with monomorphic nuclei of finely granular chromatin and amphiphilic cytoplasm of undefined edges. There were arranged delimiting nests and trabeculae, without necrosis, mitosis, or significant atypia. The bronchial lesion respected the mucosal surface of the bronchus and was 1 cm from the surgical margin of resection. Lymphatic vascular invasion was identified.

The rest of the parenchyma presented multiple nodular lesions of 1 mm to 3 mm in diameter constituted by the same cellularity of neuroendocrine habit. Both the tumor nodules described and the hyperplasia foci show positivity for TTF1 and CD56, as well as positivity for chromogranin, synaptophysin, and proliferative activity with Ki67 of 0%. Up to eight lobar and segmental nodes were isolated, three of them with neuroendocrine tumor metastasis.

With the diagnosis of DIPNECH with a typical multifocal pulmonary carcinoid tumor with R0 resection in January 2014 (mpT1N1 pathological staging), clinical-radiological follow-up was initiated. The last revision was made in June 2019 with the stabilization of interstitial lung involvement. Cg A and u 5-HIAA were within the range of normality. The patient continues with persistent, irritative dry cough with moderate effort dyspnea (basal oxygen saturation of 96%).

Case 2

A 55-year-old female was referred in January 2012 to our pneumology department in the context of a dry, irritative cough that did not improve with drugs and without seasonal variation. She was a former smoker for three years of 20 cigarettes/day and suffered chronic obstructive pulmonary disease (COPD). There were alterations in the pulmonary function test (FEV1 42%, FVC 48%, and DLCO 32%) and in the physical examination (global hypoventilation and roncus).

Chest CT showed a bilateral mosaic pattern and subcentimeter bilateral lung nodules (less than 6 mm) and calcified linear tracts in the pulmonary vertices and the apical segment of the lower left lobe (Figure 1B). Bronchoscopy did not reveal any endobronchial finding. CgA, u 5-HIAA, and Octreoscan were normal. A surgical biopsy was performed. The histological surgical specimen showed pulmonary parenchyma with the presence of a carcinoid tumor, tumorlet, and hyperplasia of neuroendocrine cells. Immunohistochemistry was positive for CD56, TTF1, and CK7 and negative for CK20, HMB45, and EMA; and the proliferative index Ki67 (MID1) was 3%.

In November 2014, she received treatment with radiosurgery for venous hemangioma of the left cavernous sinus and in 2015, was diagnosed with Hashimoto's thyroiditis. The last revision was made in February 2019, with stabilization of interstitial lung involvement and normal levels of Cg A and u 5-HIAA. She continues with chronic cough without dyspnea.

Case 3

**Figure 2:**
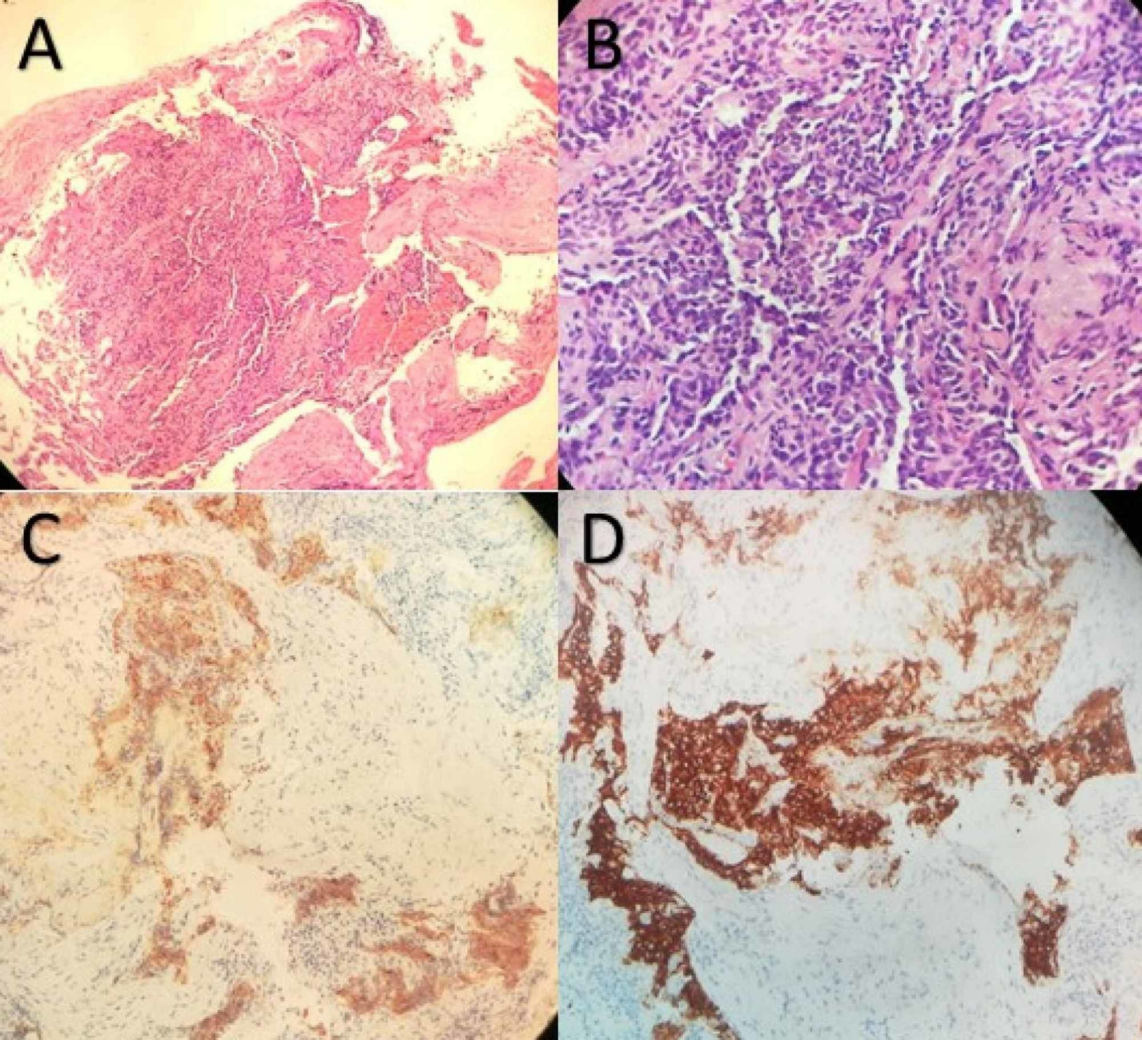
A.Tumoral cells HE stain 100x. B. Cells details HE stain 400x. C. Neoplastic cells stained with chromogranin A (200x). D. Neoplastic cells stained with Synaptofisin (200x) Histologic sections showed a small nodule (< 0.5 cm) composed of a relatively uniform population of cells with oval or spindle nuclei. The tumor cells were arranged in nests and cords with finely granular chromatin, inconspicuous nucleoli, and moderate amounts of clear cytoplasm. Mitotic activity and necrosis were absent. There was no evidence of airway inflammation, interstitial fibrosis, and remodeling of vascular structure in the remaining lung tissue. Neuroendocrine markers, such as chromogranin A, synaptophysin, neuron-specific enolase (NSE), and CD56, were strongly positive in these tumoral cells. We diagnosed it as carcinoid tumorlet.

A 60-year-old female was referred in May 2014 to our pneumology department in the context of a history of more than 30 years of evolution with dry cough and exertional dyspnea for two years. She was a nonsmoker. The chest radiograph showed a bilateral micronodular pattern with a paracardiac nodular image in the right lower lobe (RLL). She is the sister of the patient in Case 2. The pulmonary function test was altered (FEV1 27%, FVC 52%, and DLCO 49%). CT showed bilateral apical pleural thickenings and opacity with loss of volume in the middle lobe (ML), with multiple pulmonary nodules in both lobes up to 11 mm (Figure 1C). Bronchoscopy did not show any finding, but a transbronchial biopsy was performed on RLL and ML. The definitive pathology results are summarized in Figure 2.

**Figure 3:**
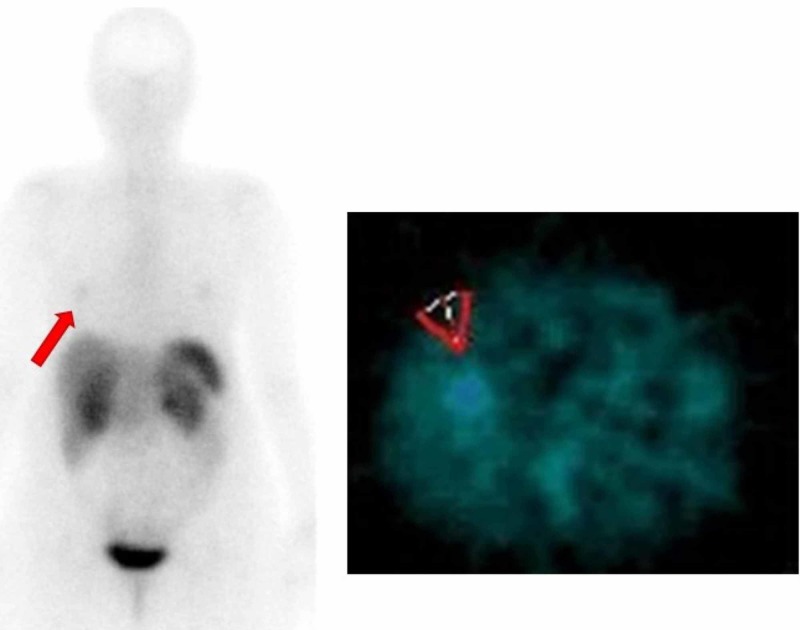
Showing slight metabolic activity located in the basal medial segment of the right inferior pulmonary lobe (red arrows) in a probable relationship with viable tumor lesion expressing (mild grade) somatostatin receptors (rSS-2 and rSS-5)

The study was completed with a PET-CT (pathological uptake in the ML lesion with SUV max of 3.87 and uptake of the rest of the bilateral pulmonary nodules with SUV max of 3.52), Octreoscan (with pathologic uptake in ML, Figure 3), plasma CgA (normal), and u-5-HIAA (high levels of 25 micrograms in 24 hours). Despite the elevated levels of u-5-HIAA, the patient didn´t have clinical signs compatible with carcinoid syndrome. The study was extended with an echocardiogram that was normal.

In the context of uncontrolled cough with habitual medication, positive Octreoscan, and elevated levels of u-5-HIAA, it was decided to start treatment with lanreotide depot 120 mg subcutaneously every 28 days. During the first three months, cough improvement and decrease of u 5-HIAA to 14 micrograms in 24 hours were achieved. Grade 2 diarrhea, abdominal pain, and headache presented as side effects of the lanreotide treatment, so the dose was lowered to 90 mg every 28 days with a slight improvement in tolerability. After nine months of treatment, the lanreotide was discontinued at the request of the patient. There was no improvement in the pulmonary interstitial involvement in the posterior CT scans. The Octreoscan was repeated one year after the end of the lanreotide, with negativization of the pulmonary uptake. She continued with chronic cough and moderate effort dyspnea. The CT scan in April 2019 showed stabilization of the interstitial lung involvement. Cg A was negative and u-5-HIAA was 12 micrograms in 24 hours.

Case 4

A 67-year-old female was evaluated in November 2015 in the pneumology department for dyspnea of years of evolution and chest tightness. She was a non-smoker, suffered obesity and osteoporosis, and had surgical resection for hyperparathyroidism. Thoracic CT showed linear and reticular opacities, some of them tending to coalesce and consolidate, with a predominantly peripheral distribution (Figure 1D). Bronchoscopy provided no significant information but a transbronchial biopsy was performed on RLL and ML. The pathology results were multifocal, micronodular, with linear cell proliferation, immunohistochemistry (Synaptophysin +) and morphologically suggestive of neuroendocrine hyperplasia, limited to the bronchiolar epithelium and peripheral peri-adjacent pulmonary parenchyma, which respects the basal membrane of the remitted respiratory epithelium. There were alterations in the pulmonary function test (FEV1 44%, FVC 65%, and DLCO 29%). CgA, u-5-HIAA, and Octreoscan were normal.

About three months later, the patient started with pyrosis, postprandial pain, anorexia, and weight loss. An endoscopy and new body CT revealed a stenosing pyloric neoplasm. Biopsies confirmed the diagnosis of HER2-negative gastric adenocarcinoma. The staging was uT3 N1 Mx. She started neoadjuvant chemotherapy treatment with the EOX scheme (epirubicin, oxaliplatin, and capecitabine). She was admitted to the intensive care unit after two weeks (February 2016) due to influenza A virus bilateral pneumonia and died days later due to respiratory distress syndrome.

## Discussion

DIPNECH is an entity that becomes more common in the literature every year [4]. Most patients are females between the fifth and sixth decades, non-smokers, with obstructive lung symptoms and peripheral lesions [5]. The demographics and presentation of disease in our cases support the current literature with the exception of two patients (50%) who were smokers/former smokers (Table 1).

**Table 1:** Clinical characteristics of the four index patients with DIPNECH DIPNECH, diffuse idiopathic pulmonary neuroendocrine cell hyperplasia; COPD, chronic obstructive pulmonary disease

Patient	Sex	Age	Smoking status	Symptoms	Duration of symptoms	Imaging findings	Type of lesion
1	Female	48	Smoker	Chronic cough and dyspnea.	Multiple years	Multiple bilateral nodules	Tumorlets + Carcinoid
2	Female	55	Former smoker	COPD and chronic cough	10 years	Multiple bilateral nodules	Tumorlets + Carcinoid
3	Female	60	No smoker	Chronic cough and dyspnea.	>30 years	Bilateral apical pleural thickenings and multiple bilateral nodules	Tumorlets
4	Female	67	No smoker	Dyspnea and chest tightness	Multiple years	Bronchiectasis and opacities	Tumorlets Lung + Gastric Adenocarcinoma

The classical histopathological features of DIPNECH include a widespread proliferation of small bland uniform cells within the epithelium, showing a lack of mitoses or necrosis, accompanied by neuroendocrine features, such as the fine soft chromatin pattern and immunoreactivity to neuroendocrine markers [6]. A pathology-based approach by Marchevsky et al. [7] aimed at distinguishing DIPNECH from reactive neuroendocrine cell hyperplasia (NECH) suggested that the presence of multifocal NECH associated with ≥ three tumorlets could represent a pathological criterion for the diagnosis of DIPNECH.

Although in our series, there is a family association of two sisters affected by DIPNECH, there is no clear evidence of family aggregation/germline genetic alterations in this entity. The possible association between DIPNECH and pulmonary adenocarcinoma is described [8], but not with other types of tumors. However, two of our patients (50%) have a metachronous tumor (perianal squamous cell carcinoma and gastric adenocarcinoma).

DIPNECH is usually diagnosed as an incidental finding in asymptomatic patients without radiological abnormalities [3]. However, patients usually show attenuated symptoms of years of evolution, such as exertional dyspnea, wheezing, and dry cough. All of our patients had clinical symptoms (cough and dyspnea) of years of evolution.

Careful integration of clinical, functional, and imaging data, along with the histological demonstration of constrictive bronchiolitis akin to neuroendocrine cell proliferation, is mandatory to establish a diagnosis of DIPNECH [2].

Treatment and prognosis depend on the severity of constrictive obliterative bronchiolitis. In our series, 100% of cases (4/4) were symptomatic, mainly due to cough and exertional dyspnea. Long-term follow-up is recommended to exclude nodular growth and the development of carcinoid tumors or invasive carcinoma. Two patients had a concomitant carcinoid tumor. Three patients kept the disease stable during follow-up (median follow-up of 74 months) while one patient died six months after diagnosis due to metachronous gastric adenocarcinoma. In addition, the disorder can progress to severe airway obstruction, and several deaths have been reported from the progressive decline of pulmonary function associated with pulmonary fibrosis [9].

Limited data are available regarding the use of somatostatin analogs (SSA) in DIPNECH. Gorshtein et al., in their review of 11 DIPNECH patients, suggested the affirmative role of SSA in the symptom management of DIPNECH [5]. In the American single-center experience, most of their patients responded to treatment with SSA and had significant improvement in their presenting symptoms [10].

In one patient, we currently use lanreotide depot 120 mg deep subcutaneous injection every 28 days. There is no evidence that SSA will retard growth or transformation to carcinoid; however, treatment can be lifelong if patients respond favorably without adverse side effects to SSA. Although significant cough improvement was obtained, dyspnea remained stable and mild side effects appeared (diarrhea and headache), so the treatment was interrupted.

## Conclusions

In summary, DIPNECH represents a rare and poorly understood pulmonary disorder that includes incidental cases of neuroendocrine cell proliferation observed in the context of various pulmonary disease forms associated with carcinoid tumors. It affects females over 60 years of age, and dry cough is the most common presenting symptom. Our limited single-center experience shows the slow and stable evolution of the disease. SSA can be useful for the management of symptoms. However, some exceptional cases may progress poorly if distant metastases or progressive decline of pulmonary function occur.

## References

[1] Aguayo SM, Miller YE, Waldron JA (1992). Brief report: idiopathic diffuse hyperplasia of pulmonary neuroendocrine cells and airway disease. N Engl J Med.

[2] Rossi G, Cavazza A, Spagnolo P (2016). Diffuse idiopathic pulmonary neuroendocrine cell hyperplasia syndrome. Eur Respir J.

[3] Davies SJ, Gosney JR, Hansell DM (2007). Diffuse idiopathic pulmonary neuroendocrine cell hyperplasia: an under-recognised spectrum of disease. Thorax.

[4] Wirtschafter E, Walts AE, Liu ST, Marchevsky AM (2015). Diffuse idiopathic pulmonary neuroendocrine cell hyperplasia of the Lung (DIPNECH): current best evidence. Lung.

[5] Gorshtein A, Gross DJ, Barak D, Strenov Y, Refaeli Y, Shimon I, Grozinsky‐Glasberg S (2012). Diffuse idiopathic pulmonary neuroendocrine cell hyperplasia and the associated lung neuroendocrine tumors: clinical experience with a rare entity. Cancer.

[6] Ge Y, Eltorky MA, Ernst RD, Castro CY (2007). Diffuse idiopathic pulmonary neuroendocrine cell hyperplasia. Ann Diagn Pathol.

[7] Marchevsky AM, Wirtschafter E, Walts AE (2015). The spectrum of changes in adults with multifocal pulmonary neuroendocrine proliferations: what is the minimum set of pathologic criteria to diagnose DIPNECH?. Hum Pathol.

[8] Gorospe L, Muñoz-Molina GM, Farfán-Leal FE, Cabañero-Sánchez A, García-Gómez-Muriel I, Benito-Berlinches A (2017). Association of diffuse idiopathic pulmonary neuroendocrine cell hyperplasia (DIPNECH) with lung adenocarcinoma: a radiologist's perspective. Lung Cancer.

[9] Klebe S, Henderson DW (2013). Facts and fiction: premalignant lesions of lung tissues. Pathology.

[10] Chauhan A, Ramirez RA (2015). Diffuse idiopathic pulmonary neuroendocrine cell hyperplasia (DIPNECH) and the role of somatostatin analogs: a case series. Lung.

